# Explainable AI for Interpretation of Ovarian Tumor Classification Using Enhanced ResNet50

**DOI:** 10.3390/diagnostics14141567

**Published:** 2024-07-19

**Authors:** Srirupa Guha, Ashwini Kodipalli, Steven L. Fernandes, Santosh Dasar

**Affiliations:** 1 Department of Computer Science and Engineering, National Institute of Technology Durgapur, Durgapur 713209, India; 2 Department of Artificial Intelligence and Data Science, Global Academy of Technology, Bengaluru 560098, India; dr.ashwini.k@gat.ac.in; 3 Department of Computer Science, Design, Journalism, Creighton University, Omaha, NE 68178, USA; 4 Department of Radiology, SDM College of Medical Sciences Dharwad, Dharwad 580009, India; dr.santoshdasar@sdmuniversity.edu.in

**Keywords:** custom ResNet50, explainable AI methods, LIME, saliency map, occlusion analysis, GRAD-CAM, SHAP, SmoothGrad, interpretable AI

## Abstract

Deep learning architectures like ResNet and Inception have produced accurate predictions for classifying benign and malignant tumors in the healthcare domain. This enables healthcare institutions to make data-driven decisions and potentially enable early detection of malignancy by employing computer-vision-based deep learning algorithms. These CNN algorithms, in addition to requiring huge amounts of data, can identify higher- and lower-level features that are significant while classifying tumors into benign or malignant. However, the existing literature is limited in terms of the explainability of the resultant classification, and identifying the exact features that are of importance, which is essential in the decision-making process for healthcare practitioners. Thus, the motivation of this work is to implement a custom classifier on the ovarian tumor dataset, which exhibits high classification performance and subsequently interpret the classification results qualitatively, using various Explainable AI methods, to identify which pixels or regions of interest are given highest importance by the model for classification. The dataset comprises CT scanned images of ovarian tumors taken from to the axial, saggital and coronal planes. State-of-the-art architectures, including a modified ResNet50 derived from the standard pre-trained ResNet50, are implemented in the paper. When compared to the existing state-of-the-art techniques, the proposed modified ResNet50 exhibited a classification accuracy of 97.5 % on the test dataset without increasing the the complexity of the architecture. The results then were carried for interpretation using several explainable AI techniques. The results show that the shape and localized nature of the tumors play important roles for qualitatively determining the ability of the tumor to metastasize and thereafter to be classified as benign or malignant.

## 1. Introduction

Ovarian cancer is one of the most common types of cancer in women all around the world [1]. Early detection remains one of the best possible means of cure. Due to recent advancements in the Computer Vision domain, healthcare experts are leveraging CNN-based architectures to classify tumors into benign or malignant based on MRI or CT scanned images, thus enabling them to make early diagnosis of the disease. In addition to domain knowledge, experts can now rely on the capability of artificial intelligence to detect signs of cancer at an early stage, which would potentially transform the diagnosis of various types of cancer, with ovarian cancer being the most prominent [2].

The leverage of AI in the healthcare domain is increasing by the day, and healthcare institutions are relying on the usage of Computer Vision-based classification and detection deep learning algorithms for early diagnosis of diseases like cancer, which is not intuitive to predict by human professionals. This can potentially revolutionize the healthcare and disease prediction domain, and save or prolong countless lives through early diagnosis and treatment for fatal diseases like cancer. Deep learning convolutional neural network-based algorithms have proven to be one of the most accurate and efficient methods of detection of tumors from CT scans or MRI scan images. However, due to the black-box nature of these algorithms, it is difficult to interpret the results of the algorithm and understand why the model predicted a certain class. Retracing the results of the algorithm back to the individual layers can be a challenging task, and it is important to understand why a neural network made a certain prediction, or, in other words, which features it relied on the most for the final predicted output. The traditional Machine Learning algorithms like Support Vector Machines and Decision Trees provide explainability; however, the problems of image classification and localization can be solved with a greater accuracy and efficiency with deep convolutional neural networks. Sometimes, due to a class imbalance in the dataset, the network can be inclined towards a certain output and place importance on the wrong features. Hence, in this scenario, without some explainability methods, it would be difficult to rely on these algorithms. Class activation maps and feature importance techniques prove to be excellent for highlighting the regions of interest and, thus, ensuring that the algorithm results are fair and unbiased, as well as take into consideration the right features for prediction. This is crucial for healthcare practitioners to continue to rely on and have faith in deep learning algorithms to help in their decision-making processes. Thus, to interpret the results of these models, explainable AI methods are being implemented. Inspecting intermediate features at each layer is one of the popular methods to interpret model representation at various layers. The second approach is attention-based, which highlights the input features that are given most importance during prediction. Explainable AI methods, on the other hand, help to analyze the decision making process of neural networks with the help of class activation maps and local explanations, and have been implemented this paper. In some binary or multiclass classification problems, there also a scope of creating an ensemble of neural networks combined with decision tree or rule based approaches to enhance the interpretability of predictions. Finally, introducing dropout or regularization in networks, and thus increasing the sparsity of the networks, can help with identifying the features that are most important towards the final predicted output.

Recent advancements in deep learning convolutional neural network-based architectures have proven to be efficient in identifying higher- and lower-level features during classification of tumors into benign and malignant categories at earlier stages of detection, which is nearly impossible to predict with the human eye [3]. These architectures provide enhanced performance when compared to traditional Machine Learning classification algorithms like Support Vector Machines and Logistic Regression due to the consumption of huge amounts of training data and the complexity of the network architecture. However, unlike traditional Machine Learning algorithms like Logistic Regression and Decision Trees, the deep learning CNN architectures provide less explainability and interpretability of the results, despite outperforming these algorithms manyfold in terms of classification accuracy [4]. Hence, it is crucial to understand the way CNN algorithms work for making a classification and the importance of the features considered for the final classification result for the healthcare experts to make a data-driven diagnosis with more confidence and prescribe the most effective treatment [5].

In this paper, we have developed a ResNet60 model, which is a modified ResNet50 model derived from the standard ResNet50, for the classification of ovarian tumors into benign and malignant. The modified ResNet50 model is derived by adding more Convolutional layers to ResNet50, resulting in a ResNet60 model, and the detailed architecture is discussed in the methodology section of the paper. The motivation behind designing a custom ResNet50 was to attain higher classification performance while ensuring that the complexity of the network does not increase by adding more layers, as in ResNet101 or ResNet151, which could lead to vanishing and exploding gradient problems during training. The derived ResNet50 showed higher classification performance than the standard ResNet50 by an increased 7.5% on the test dataset without increasing the complexity of the network to a large extent. This model was also compared with several state-of-the-art architectures, and has outperformed the other architectures with a classification accuracy of 97.50% on the ovarian tumor test dataset. Therefore, the results of the derived ResNet50 classifier are carried forward for interpretability.

To explain the results of the modified ResNet50 classifier, we have implemented the following explainable AI methods: LIME, Saliency Map, Occlusion Analysis, Grad-CAM, SHAP, SmoothGrad. The Explainable AI methods used in this paper are the ones that are most commonly used in existing literature related to Explainable AI for a qualitative interpretation of the model results.

LIME is a model-agnostic method that aims to explain the important features behind the classification results by creating several artificial data samples and thereby predicting the class of each such artificial data point. The Saliency Map highlights the pixels that most affect the class scores. Occlusion analysis evaluates the importance of each input dimension by analyzing the model performance with that input dimension missing and its impact on the model performance. Grad-CAM weighs the gradient in the final CNN layer against the output of this layer to determine which features are the determining factors behind the class predicted. SHAP is a feature importance technique, while SmoothGrad adds a small Gaussian noise and highlights the important pixels contributing to the final predicted class across all the training images. Using these explainable AI methods, we interpret and explain the results of the modified ResNet50 by highlighting the important features and pixels considered by the classifier while classifying ovarian tumors into benign and malignant. Furthermore, it helps us understand the key characteristics of benign ovarian tumors, which distinguish them from malignant tumors.

The main contributions of this paper are summarized below:

- A modified custom ResNet50 (or, in terms of convolutional layers, a ResNet60) architecture is proposed for the classification of ovarian tumors as benign and malignant. The modified ResNet50 classifier performs the classification task with 97.5% accuracy on the test dataset, which is higher than other state-of-the-art architectures implemented—GoogLeNet (Inception-v1), Inception-v4, ResNeXt, Inception-ResNet, ResNet16, Xception, VGG16, VGG19, ResNet50, EfficientNetB0.
- The results of the modified ResNet50 are then provided for interpretation to the explainable AI methods LIME, Saliency Map, Grad-CAM, Occlusion Analysis, SHAP and SmoothGrad. In order to have a meaningful and accurate interpretation of the classification output, the explainable AI methods were implemented on the classification results of the modified ResNet50 architecture, since it had the best classification accuracy on the test dataset.
- The above explainable AI methods highlighted the features and regions of interest that were given most importance by the proposed model in obtaining the final classification output, such as the shape and neighboring area of the tumor. The results show qualitatively that the tumors that have a fixed boundary and localized nature are classified as benign by the proposed model, while those with an irregular shape and boundary are classified as malignant by the model, indicating that the localization, boundary, and shape are important characteristics to understand qualitatively from the image as to whether the tumor has a tendency to metastasize to neighboring organs and tissues, subsequently leading to the benign or malignant nature.

The organization of the paper is as follows: Section 2 presents an overview of the related work in tumor classification and various explainable AI approaches like LIME, SHAP, Grad-CAM, Occlusion Analysis, SmoothGrad for image classification in general, as well as in the medical imaging domain. Section 3 discusses the gap in the existing literature for leveraging explainable AI methods used in classifying ovarian tumor images and the motivation behind this work. Section 4 discusses the proposed methodology. Section 5 describes the results of the proposed ResNet60 (modified ResNet50) architecture and an overview of the explainable AI methods used for the interpretation of the results. It also presents a comparison of the performance of the proposed custom ResNet50 with state-of-the-art architectures, and shows that the proposed architecture yields the highest test accuracy in the classification of tumors and, hence, the results are interpreted further using the explainable AI methods as mentioned above. Finally, it discusses the scope of future work in this domain. Section 6 discusses the conclusion, along with the key takeaways from this research work.

## 2. Related Work

### 2.1. Existing Work Explainability of Classification Models in the Medical Imaging Domain

In the healthcare domain, the use of AI to classify images of tumors, obtained from CT scans or MRI scans, as benign or malignant is increasingly becoming a useful tool for the early diagnosis of cancer. Also, with the advent of COVID-19, we see research work on the classification of chest X-ray images for presence or absence of COVID-19 or pneumonia. Some of these use cases are illustrated by the following research papers. For example, Manali Gupta et al. [6] implemented and evaluated the performance of a scratch CNN method with VGG-16 for classification of brain tumor MRI images as cancerous or non-cancerous. Samir S. Yadav et al. [7] evaluated convolutional neural network-based architectures for the classification of pneumonia presence on a chest X-ray images dataset. Although deep neural networks have proved to be effective in the medical image classification task, with an increasing number of layers, the training process can slow down and become less effective due to the problems of a vanishing and exploding gradient. This problem is solved by introducing a residual network, commonly known as ResNet, which enhances the training of deep neural networks. Jiazhi Liang et al. [8], in their research paper, illustrated the ResNet architecture for image classification using deep neural network models.

### 2.2. Existing Work on Explainability of CNN Models in the Medical Imaging Domain

Post training, it is important to interpret the CNN classification output, which is difficult due to the black box nature of these deep learning algorithms. Hence, explainable AI methods are crucial at this point to interpret and explain the classification predictions obtained from the neural network. The following are some surveys and review papers on explainable AI methods, used for reference. Saranya, A. et al. [9] discussed some of the commonly used explainable AI methods like LIME and SHAP, and several versions of the same. Baehrens, D. et al. [10] used local explanation vectors for explaining classification results. Xu, F. et al. [11] performed a detailed survey of the history and various methods in the field of explainable AI. Yang, W. et al. [12] discussed various explainable AI approaches, along with their limitations and use cases. Samek, W. et al. [13] presented recent developments in the field of explainable AI and discussed two specific methods for the same. Singh, A. et al. [14] discussed the explainable AI methods in the medical image analysis domain. Velden, B.H.M. et al. [15] presented a survey report of existing explainable AI techniques in the deep learning-based medical image domain, and discussed future prospects for the same. Linardatos, P. et al. [16] provided a review of various interpretability methods in the Machine Learning domain. Among the explainable AI methods, the most popular ones are LIME, Saliency Map, Occlusion Analysis, Grad-CAM, SHAP, and SmoothGrad. The following are references to the research papers proposing the above methods on various datasets for interpreting CNN classification output. Ribeiro, M.T. et al. [17] proposed the LIME technique for demonstrating the explainability of the Machine Learning classifiers. Junkang An at al. [18] propose a LIME-based explainable AI technique for interpreting the results of deep learning models using feature importance and partial dependency plots (PDPs). Alqaraawi, A. et al. [19] explored the Saliency Map method in detail using public datasets. Simonyan, K. et al. [20] used two methods, one using a class score and another using a Saliency Map, for analyzing the results of a deep CNN. Xiao-Hui Li et al. [21] evaluated and compared various explainable AI methods based on defined evaluation metrics. Resta, M et al. [22] provided an occlusion-based technique for the explanation of deep recurrent neural networks for biomedical signals. Selvaraju, R.R. et al. [23] used the Grad-CAM technique for visual explanations of the classification predictions made by deep convolutional neural networks. Cao, Q.H. et al. [24] proposes a novel explainable AI method, Segmentation - Class Activation Mapping (SeCAM), that combines the best features of LIME, CAM, and the GradCAM methods for explanation of CNN prediction results. Ruigang Fu et al. [25] proposed an axiom-based Grad-CAM to satisfy the axioms of sensitivity and conservation. Ioannis Kakogeorgiou et al. [26] evaluated various explainable AI methods in the context of deep learning multi-label classification for remote sensing. Lundberg, S. et al. [27] proposed the SHAP method for interpreting the model predictions using feature importance values for a particular prediction. Bach, S. et al. [28] described an approach based on pixel-wise contributions to explain the classification predictions made by non-linear classifiers. Hooker, S. et al. [29] evaluated the performance of feature importance estimation methods used by interpretable or explainable AI methods. Ishikawa, S. et al. [30] proposed a method of explainable artificial intelligence to verify the reliability of a deep learning model for remote sensing image classification tasks. Jogani, V. et al. [31] used various explainable AI techniques to interpret the results of CNNs for classification of lung cancer from histopathological images. Montavon, G. et al. [32] used Deep Taylor Decomposition to explain the results of non-linear classifiers. Shrikumar, A et al. [33] proposed a method, Deep Learning Important Features (DeepLIFT), which assigns contributions towards the final output to the neurons of the deep neural network and also illustrates positive and negative contributions. Smilkov, D. et al. [34] proposed a method called SmoothGrad for explaining the results of a deep neural network. Soltani, S. et al. [35] provided enhanced explainable AI algorithms based on cognitive theory. Springenberg, J.T. et al. [36] proposed a deconvolutional approach for interpreting the classification results of the CNNs. Sundararajan, M. et al. [37] proposed the Integrated Gradients approach for explaining the deep neural network predictions. Vermeire, T. et al. [38] proposed the SEDC method, which is model agnostic and is able to provide counterfactual explanations for the predictions of deep convolutional neural networks. Zeiler, M.D. et al. [39] demonstrated the performance contribution of different layers of the ImageNet used for classification. Zhou, B. et al. [40] proposed a modification of the global average pooling layer and implement the Class Activation Mapping (CAM) technique to improve the CNN’s performance in classification of the images without being trained specifically for the same. Wu, B. et al. [41] proposed and evaluated an attention-based model for large-scale image classification, and thereby explain the classification results of this approach. Szegedy, C. et al. [42] and LeCun, Y. et al. [43] discuss approaches for enhancing CNNs. A comparative analysis of the existing work with our research work is shown in Table 1 and Table 2.

## 3. Research Gap and Motivation

Deep learning models have proved to be accurate to classify tumors as benign or malignant, and there is plenty of literature demonstrating the same. However, due to the black-box nature of deep learning models, it is difficult to interpret and accurately pinpoint the features or pixels responsible for the final model output, and to distinguish one class from another. There is limited literature on developing explainable AI methods to interpret and explain the classification output of these models for better data-driven decision-making in the healthcare domain. Moreover, there is a lack of adequate research work on leveraging explainable AI techniques to interpret the results of deep learning models used for classification of tumors into benign and malignant in the healthcare domain for the healthcare experts to take better data-driven decisions using AI and thus helping in early diagnosis of the disease. The existing literature on tumor classification does not highlight the features or regions of the tumors that are considered significant by the model during classification. The lack of qualitative analysis of the model results poses a challenge in decision making due to the black-box nature of the deep learning models and their reliability, despite their high classification performance. It is essential to understand that the classifier recognizes the right patterns during classification (for example, the shape, size and other characteristics of tumors) that determine whether they have the tendency to be benign or malignant. Hence, in this paper, we use several explainable AI techniques to explain the results of a custom enhanced ResNet50 classifier that classifies the ovarian tumors as benign and malignant with 97.50% accuracy on the test dataset, and outperforms the state-of-the-art ILSVRC winning architectures. The interpretation of the results show that the shape, boundary, and ability of the tumor to be localized in nature are important factors considered by the proposed model during classification, and this qualitative analysis also supports the basic definition of benign and malignant tumors in medical science based on their ability to metastasize and spread to neighboring tissues and organs.

## 4. Methodology

**Figure 1:**
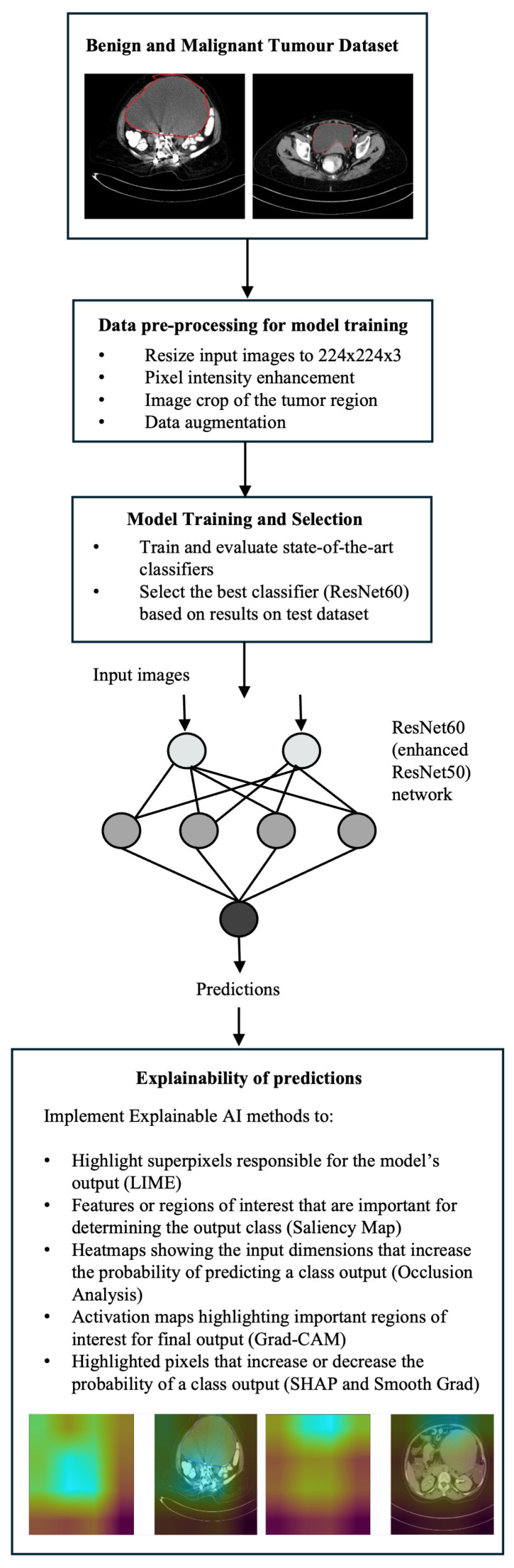
Methodology of model selection for ovarian tumor classification and qualitative explanation of results.

The proposed enhanced ResNet50 model has been trained on the dataset of cropped ovarian tumor images for the classification of the tumors as benign or malignant. The training dataset comprises 3644 images, with 2633 belonging to benign and 1011 belonging to the malignant classes. The model has been validated on a dataset of 1561 images, comprising 1128 benign images and 433 malignant images. The model has also been tested using a test dataset of 520 images, comprising 376 benign and 144 malignant images, on which it achieved an accuracy of 97.50% on the test dataset. There is a class imbalance in the datasets, where benign images are higher in number than malignant images. Figure 1 shows the methodology implemented.

### 4.1. Enhanced ResNet50 (Proposed ResNet60) Architecture

**Figure 2:**
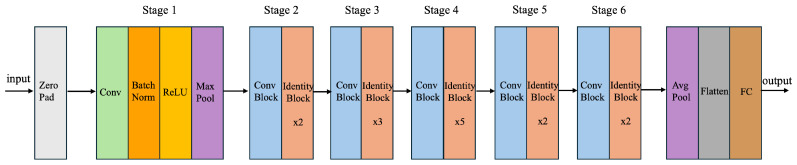
ResNet60 (enhanced ResNet50) architecture for classification of ovarian tumor images into benign and malignant.

The proposed architecture considers inputs images of size 224 × 224 × 3, and the various layers are shown in Figure 2.

**Figure 3:**
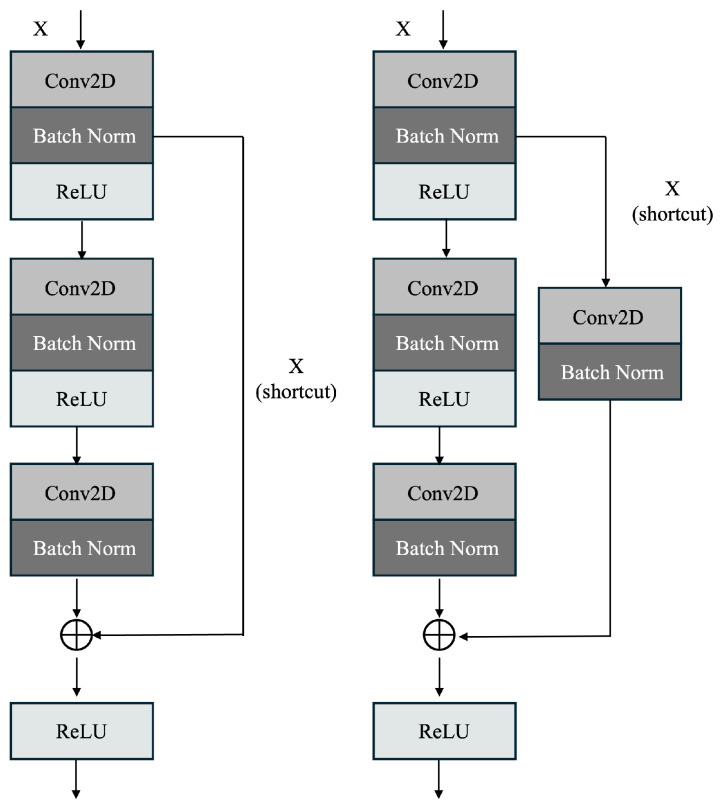
Identity block (**left**) and Convolution block (**right**) for enhanced ResNet50 architecture.

**Figure 4:**
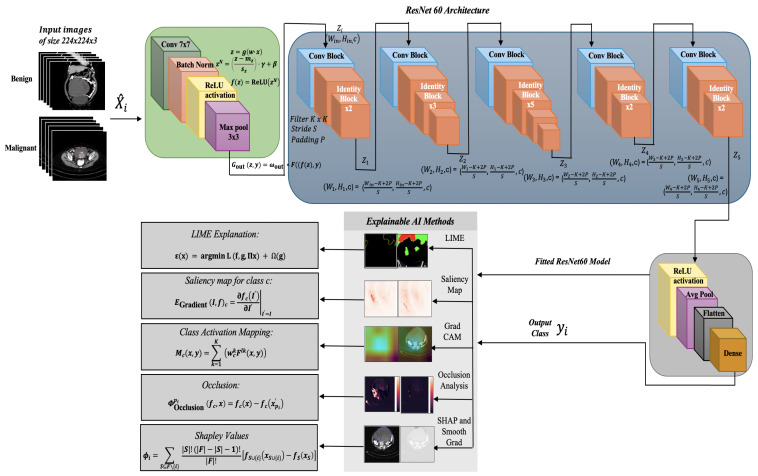
Proposed system comprising the enhanced ResNet50 (or ResNet60) architecture in detail and Explainable AI methods.

While the complete architecture is shown in Figure 2, the structure of each convolutional and identity block is displayed in Figure 3. Figure 4 shows the proposed system comprising the various layers of the ResNet60 architecture, obtained by adding additional layers to a pretrained ResNet50, followed by the model explanations.

As the deep learning architectures continued to add more layers for higher accuracy of the predicted output, the vanishing and exploding gradient problems started becoming more common with increasingly deep layers, thereby slowing down the learning and reducing the accuracy of the predicted output. This problem was solved by ResNet, where residual blocks containing skip connections were introduced, which connect activations of a layer to deeper layers, skipping some layers in between.

The fundamental building blocks of the ResNet architecture and image classification is described in Algorithm 1.

To interpret the classification results of the modified ResNet50 model, we have implemented the above-mentioned explainable AI methods. The objective of this research is to determine the important features or pixels responsible for the proposed system to produce the final classification output. We also aimed to understand what features distinguish a benign ovarian tumor from a malignant ovarian tumor based on the CT scanned image, and how the neural network captures the same.

**Algorithm 1:** Image classification using enhanced ResNet50 architecture.

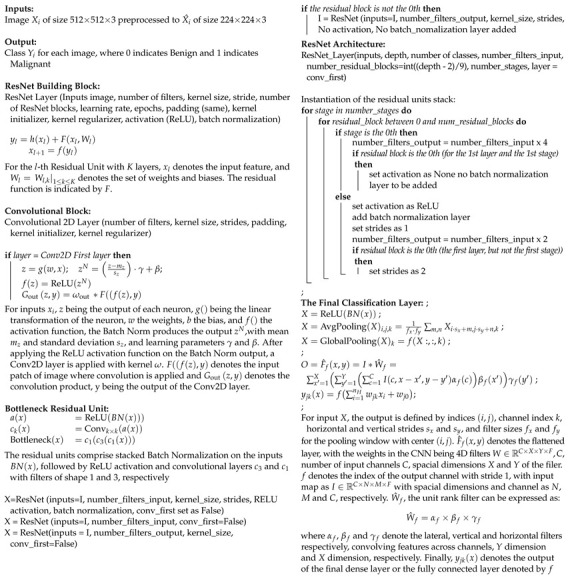



### 4.2. Explainable AI Methods

To interpret the classification results of the enhanced ResNet50 model, we have implemented the above-mentioned explainable AI methods. The objective of this research is to determine the important features or pixels responsible for the proposed model to produce the final classification output. We also aimed to understand what features distinguish a benign ovarian tumor from a malignant ovarian tumor based on the CT scanned image, and how the neural network captures the same.

LIME, which stands for Local Interpretable Model-agnostic Explanations, is an Explainable AI method that aims to explain the results of a model using a local linear approach. LIME creates several artificial data points in the vicinity of the data point, whose classification results it aims to explain. It uses a local linear classifier to predict the class of each of the artificially created data points. After this, the cosine similarity is calculated between each artificial data point and the original data point to determine the importance or weight of each of the artificial data points in classifying the real data point. Since the dataset consists of images, the data points are pixels, and the cosine distance is computed between the pixels to compute their weight in determining the class. Using the artificially weighted data points, a linear regression model is fitted, and the features or data points with the higher coefficients in the fitted linear regression model contribute the most in determining the prediction of the model being evaluated.

Riberio et al. [17] described a LIME-generated explanation as:

$$\varepsilon \left(x\right)=\mathrm{argmin}L\left(f,g,\Pi_{x}\right)+\Omega \left(g\right)$$

where *g* indicates the model used for approximating the function *f*, $\Pi_{x}$ indicates the distance measured between *z* and the neighborhood around *x*, with *z* and *x* being instances. The complexity of explanation of model outputs using *g* is depicted by Ω(g).

While LIME uses feature importance to explain the importance of nearby pixels, the Saliency Map is another explainable AI method to explain the classification results of Computer Vision models. The Saliency Map highlights the region of interest, comprising the pixels that are the determining factors behind the model’s output. The Saliency Map calculates the derivative of the class score with respect to the image, and helps us identify which pixels, when changed, cause the least change in the class score.

Simonyan et al. [20] has proposed the gradient of the output class calculated with respect to the input pixels of the image. The Saliency Map for a class c can be described as:

$$E_{\mathrm{Gradient}\;}{\left(I,f\right)}_{c}={\left.\frac{\partial f_{c}\left(I^{\prime }\right)}{\partial I^{\prime }}\right|}_{I^{\prime }=I}$$

$f_{c}\left(I^{\prime }\right)$ can be approximated with a linear function in the neighborhood of *I* by the first-order Taylor expansion: $f_{c}\left(I^{\prime }\right)\approx E{\left(I,f\right)}_{c}^{I}\cdot \left(I^{\prime }-I\right)+b$. The weights of this approximated linear function are the generated Saliency Map.

Occlusion Analysis is an attribution technique that determines the importance of each feature or dimension by evaluating the model performance by having that dimension missing. The region or patch of pixels which, when dropped, causing the highest drop in the model’s performance tends to have the most contribution towards the prediction results of the model.

Loannis Kakogeorgiou et al. [26] describe occlusion as:

$$\Phi_{Occlusion}^{p_{i}}\left(f_{c},x\right)=f_{c}\left(x\right)-f_{c}\left(x_{p_{i}}^{\prime }\right)$$

This technique calculates the change in the approximated function $f_{c}\left(x\right)$ by replaced contiguous rectangular patches $p_{i}\in P$ of the input image with a given baseline (e.g., all-zero patch).

Grad-CAM, standing for Gradient Class Activation Map, is an Explainable AI technique that produces a heatmap that shows the importance of each of the classes for the model being evaluated. To achieve this, the gradient with respect to the final layer of the CNN is computed and weighed against the output class of that layer. The heatmap is essentially a spatial map of the weight or importance of each channel towards the final output class.

Ruigang Fu et al. [25] describe the general form of the Class Activation Mapping (CAM) as:

$$M_{c}\left(x,y\right)=\sum_{k=1}^{K}\left(w_{c}^{k}F^{lk}\left(x,y\right)\right)$$

where *c* denotes the class *c* of the input image and the CAM can be expressed as a linear combination of the features in the target layer.

The SHAPley Additive Explanation (SHAP) is an Explainable AI method that calculates the feature importance based on SHAPley values derived from cooperative game theory. The feature importance calculated using the SHAP value indicates the magnitude as well as the extent of the positive or negative effect of the feature on the final class predicted by the model, and also takes into account the presence of multicollinearity.

For calculating the importance of each feature, the model is retrained on all possible subsets of features S⊆F from the set of all features *F*. The importance of each feature is thus assigned, which indicates the effect of that particular feature on the model prediction. For the $f_{S\cup \{i\}}\left(x_{S\cup \{i\}}\right)-f_{S}\left(x_{S}\right)$, two models are trained, one trained with the features being present, denoted by $f_{S\cup \{i\}}$, and another trained without including the features, denoted by $f_{S}$. For a set *S* of input features, the values are denoted by $x_{S}$. Since the effect of withdrawal of one feature depends on the subset of the other model features, the differences are calculated for all the subsets S⊆F∖{i}. Then, the SHAPley feature importance values are computed as a weighted average of the differences. Scott M. Lundberg et al. [27] describes the computation of SHAPley values as:

$$\phi_{i}=\sum_{S\subseteq F\setminus \{i\}}\frac{|S|!(|F|-|S|-1)!}{|F|!}\left[f_{S\cup \{i\}}\left(x_{S\cup \{i\}}\right)-f_{S}\left(x_{S}\right)\right]$$

SmoothGrad is an explainable AI method that is gradient-based, similar to the Saliency Map. It improves the gradient visualizations by adding a small Gaussian noise to the input image. This helps in obtaining a clean gradient visualization, free from noise, and only highlights the important pixels that are activated across all the sampled images. These important pixels determine the final classification output of the model.

Given a neural network that classifies an image into a class from a set *C* of classes, for each input image *x*, a class activation function, denoted by $S_{c}$, is calculated for each class c∈C. The final predicted class (x) is the one with the highest score defined below (Szegedy et al. [42], 2016; LeCun et al. [43], 1998):

$$\mathrm{class}\left(x\right)={\mathrm{argmax}}_{c\in C}S_{c}\left(x\right)$$

For locating the pixels in the image of higher importance, a sensitivity map, denoted by $M_{c}\left(x\right)$ is constructed as below:

$$M_{c}\left(x\right)=\partial S_{c}\left(x\right)/\partial x$$

where $M_{c}\left(x\right)$ is the derivative of $M_{c}$ with respect to *x*, the input, and $\partial S_{c}$ represents the derivative (i.e., gradient) of $S_{c}$.

The sensitivity maps are improved by using a Gaussian kernel to smoothen $\partial S_{c}$ and taking an average of the sensitivity maps of random samples in the neighborhood of an input *x*. Smilkov et al. [34] provided the below formulation for SmoothGrad:

$${\hat{M}}_{c}\left(x\right)=\frac{1}{n}\sum_{1}^{n}M_{c}\left(x+N\left(0,\sigma^{2}\right)\right)$$

where the number of samples is denoted by *n*, and the Gaussian noise with standard deviation σ is denoted by $N\left(0,\sigma^{2}\right)$.

## 5. Results and Discussion

### 5.1. Data Source and Description

**Figure 5:**
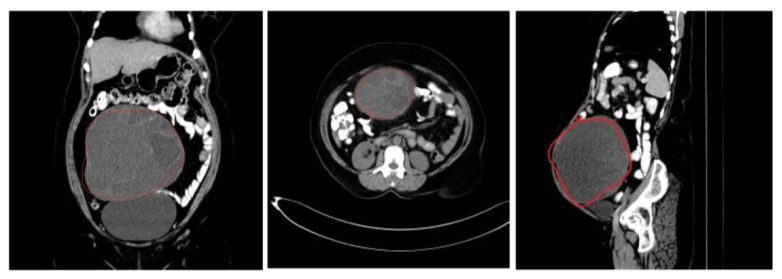
Benign ovarian tumors labeled.

**Figure 6:**
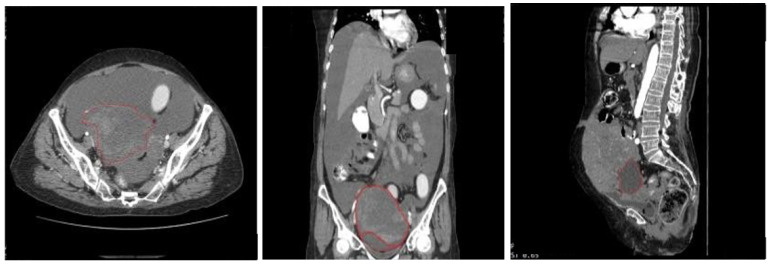
Malignant ovarian tumors labeled.

The dataset comprises a set of 5725 CT scan images of ovarian tumors, which have been annotated, that belong to benign and malignant classes for 53 unique patients from the SDM College of Medical Sciences and Hospital located in Dharwad, Karnataka, India. The dataset contains reconstructed images as per the three different planes, namely axial, sagittal, and coronal. Annotation is performed to highlight the boundaries of the tumors by encircling them with nearly-circular-shaped polygonals. Table 3 depicts the number of instances of benign and malignant images that form the training, validation, and test datasets. Figure 5 and Figure 6 show benign and malignant tumor image samples from the dataset.

### 5.2. Data Preprocessing and Dataset Preparation for Training and Evaluation

**Figure 7:**
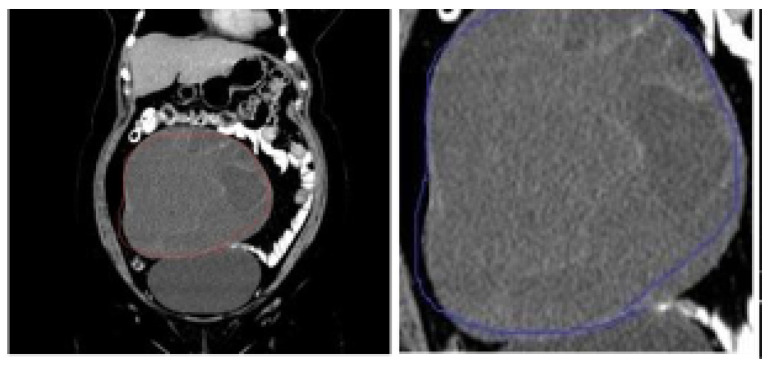
Original image in dataset (**left**) and cropped benign tumor image (**right**).

**Figure 8:**
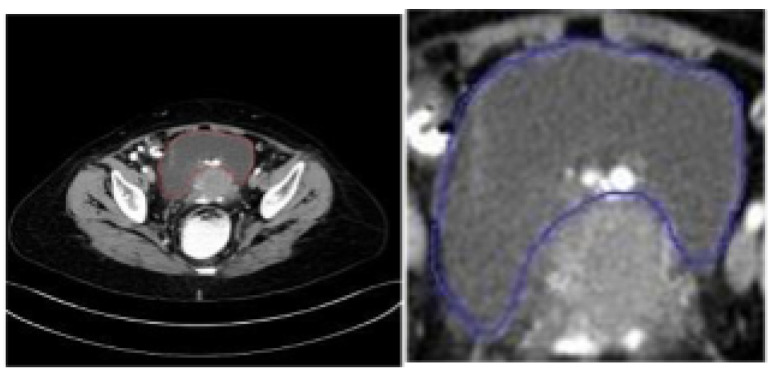
Original image in dataset (**left**) and cropped malignant tumor image (**right**).

The dataset is affected by class imbalance problem, where the benign sample instances are comparatively higher than the malignant ones. To mitigate the class imbalance issue, the images chosen for training the model were subsetted from the originally collected dataset. No specific augmentation techniques were applied to handle the class imbalance issue. For training and validation, images that have been annotated were cropped according to the annotation boundary that encircles the tumors, allowing the classification models to efficiently extract and learn the features of the localized tumor. Figure 7 and Figure 8. display illustrations of the cropping and localization applied on annotated images:

### 5.3. Classification Results

The aforementioned architectures were trained and tested on the dataset as discussed earlier. The below hyperparameters were applied to the models: input size: 224 × 224 × 3; optimizer: Adam; number of epochs for training: 200; steps per epoch: 10; validation steps: 5; loss: sparse categorical cross entropy (for inception modules) and binary cross entropy (for other modules). Table 4 shows the different layers along with filters, strides and activation functions in each layer, for the enhanced ResNet50 architecture.

Based on experiments on the train and test loss over the epochs, the learning steadily continues till 200 epochs and then slows down. On hyperparameter tuning, it was observed that the optimal number of steps per epoch that yields highest validation accuracy was 10, with a constant number of validation steps of 5. On comparing the optimizers, Adam and SGD showed the most rapid decrease in training error over the epochs, out of which Adam showed a consistent decrease beyond 150 epochs and, hence, the Adam optimizer was selected for training.

**Figure 9:**
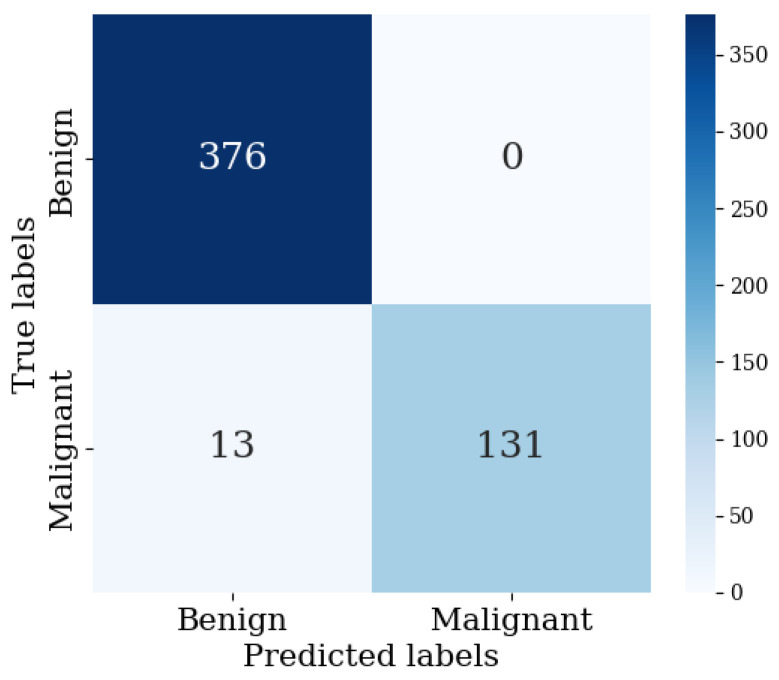
Confusion matrix for ResNet60 classification results on test dataset.

Table 5 shows the hyperparameter setting of various model architectures implemented for ovarian tumor classification. Table 6 outlines the training and test outcomes (accuracy and loss) using 200 training epochs for various architectures. After an equal number of training epochs, it is evident that the suggested ResNet60 (or enhanced ResNet50) architecture exhibits the maximum accuracy for the train as well as test datasets. In terms of accuracy, it is followed by Xception, Inception-ResNet, GoogLeNet, ResNet16, ResNeXt, and ResNet50, which exhibit test accuracy of 90% or above. During testing, an accuracy of 97.50% is obtained using the given ResNet60 model. Cross-validation of train and validation samples reveals that ResNet60 typically outperforms the other designs in terms of performance. Therefore, for the given CT scan dataset, our suggested ResNet60 design fits optimally for classifying tumors as benign or malignant. It is noted that the train and validation losses incline toward convergence, and sway about a constant value that is extremely near to zero as the epochs go by. The train and validation accuracies, at the start, show a rising trend and swing between 60 and 70%. Following 50 epochs, the accuracy rises steadily, and varies between 80 and 90%, until it stabilizes at a point where the training accuracy is between 90% and 95% and the validation accuracy is between 95% and 100%. The results demonstrate that the proposed ResNet60 has steady learning, while being free of bias and variance concerns, since it can fit the training dataset incredibly well and generalize effectively on the validation and test datasets. The confusion matrix and other detailed classification metrics for the proposed ResNet60 (modified ResNet50) model on the test dataset are shown in Figure 9 and Table 7, respectively. A precision of 96.66% indicates that, out of the total benign predictions, in 96.66% of the cases, the model was able to correctly identify it, and in only 3.34% of the predicted benign cases, it falsely predicted a malignant tumor as benign. A recall of 100% suggests that the model is able to correctly identify all benign tumors in the dataset, and there is no benign tumor falsely classified as malignant. An overall F1-Score of 98.30% suggests a good balance of the precision and recall performance. A specificity of 90.97% indicates that, out of the total number malignant tumor images in the dataset, the model was able to correctly identify the tumor in 90.97% of the cases, and in the remaining 9.03% of the actual malignant cases, the model falsely classified a malignant tumor as benign. Overall, an accuracy of 97.50% indicates that in the test dataset, the model was able to correctly predict the nature of the tumor in 97.50% of the cases.

A sample of benign and malignant images of ovarian tumors from the test dataset were fed into the below-mentioned explainable AI methods for interpretation.

### 5.4. LIME Results

**Figure 10:**
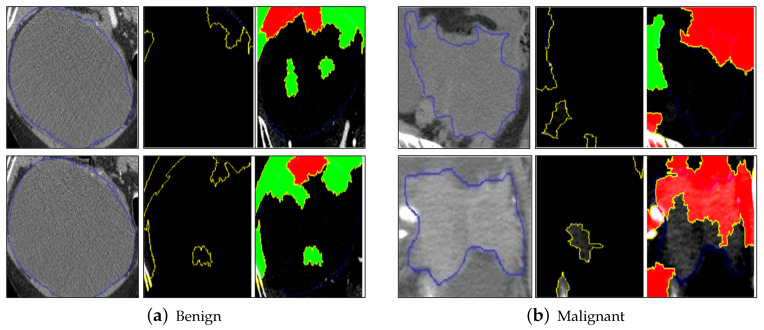
Results of LIME on benign and malignant samples of cropped ovarian tumors data. Original images (**a**) and highlighted regions with the LIME results (**b**).

The results of LIME on sample benign and malignant images are shown below in Figure 10a,b. For interpreting the results of LIME, the cropped tumor images are fed into the method, to reduce noise from the background and only focus on the data points in the vicinity of the tumor.

From the LIME results, we see why the ResNet60 model classifies the image as benign or malignant. The yellow highlighted region indicates the superpixels where the boundary of the tumor is present. This means that the model considers the pixels on the boundary of the tumor and the neighboring pixels to determine the shape and pattern of the tumor, which helps in classifying the image as benign or malignant. These super-pixels are responsible for the final classification output result for the tumor as benign or malignant. On the right image, the area of the super-pixels colored in green indicates that these pixels cause the probability of the model predicting the predicted output class (benign or malignant) increase, while the super-pixels colored in red are the ones that decrease the probability of the model predicting the final predicted output as benign or malignant. Thus, the model can identify the special features (shape, pattern, etc.) and characteristics of the tumor that are specific for the tumor to be benign or malignant, and this is highlighted by the superpixels identified by LIME, as well as the areas of the super-pixels which increase or decrease the probability of arriving at the final predicted output classification.

### 5.5. Saliency Map Results

**Figure 11:**
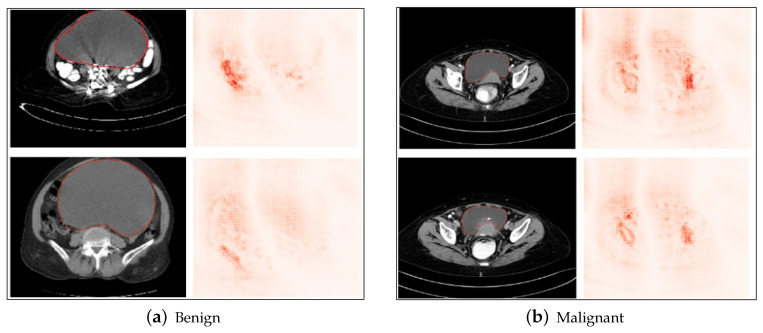
Results of Saliency Map on benign and malignant samples of cropped ovarian tumors data. Original images (**a**) and highlighted regions with the Saliency Map results (**b**).

The results of the Saliency Map as shown in Figure 11a,b show the highlighted pixels or the region of interest that are important in the final classification of the tumor into benign and malignant classes by the ResNet60 model. The calculation of saliency is similar to backpropagation. Here, the derivative of the class score is taken with respect to the image. This helps us identifying which pixels, if changed the least, affect the class scores the most.

### 5.6. Occlusion Analysis Results

**Figure 12:**
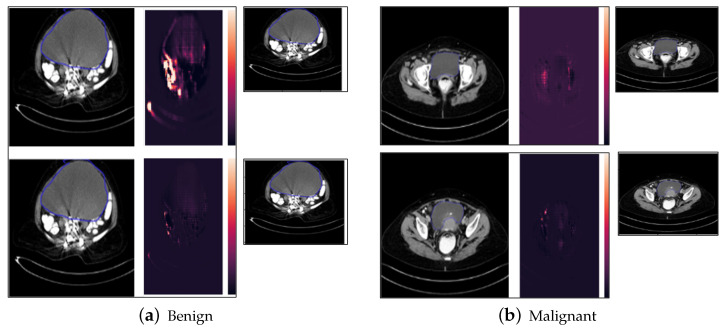
Results of Occlusion Analysis on benign and malignant samples of cropped ovarian tumors data. Original images (**a**) and highlighted pixels with the Occlusion Analysis results (**b**).

From the results of the occlusion analysis, as shown in Figure 12a,b, we observe that the highlighted regions of the heatmaps generated for the benign and malignant samples indicate the most important input dimensions towards generating the final classification, since the difference between the model outputs with and without those dimensions are higher compared to the other input dimensions. The following are the hyperparameters used for the occlusion analysis experiment towards the benign and malignant samples: occluding size: 5; occluding pixel: 0; occluding stride: 1.

We also see that the occlusion analysis method highlights the shape of the tumor and the neighboring region surrounding the tumor; hence, these are important for the final predicted output.

### 5.7. Grad-CAM Results

**Figure 13:**
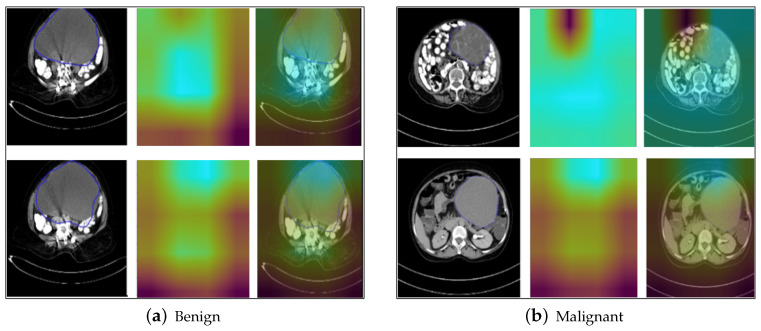
Results of Grad-CAM on benign and malignant samples of cropped ovarian tumors data. Original images (**a**) and highlighted regions with the Grad-CAM results (**b**).

From the results of Grad-CAM, as shown in Figure 13a,b, we see the heatmaps for the images of the tumor. The size of the heatmap is determined by the spatial dimensions of the activation maps in the last convolutional layer of the network. On projecting the heatmap to the original image, we see the areas of the image that are highlighted, and these areas have been assigned the most importance for the model to predict the final output (benign or malignant) for the image. It is observed that the network took the area surrounding the tumor as a determining factor for the classification of the tumor as benign and malignant. This is a reasonable explanation for the model, because the neighboring areas of the tumor indicate whether the tumor has spread to the adjacent regions, which indicates the tumor is malignant, else it is benign. Also, another characteristic of the tumor considered by the model for classification as benign or malignant is the shape of the tumor; if it has a smooth shape, which has not yet spread to the neighboring areas and is concentrated in a single region, then it is a benign tumor. Otherwise, if the tumor has an irregular shape, it indicates that the tumor has disintegrated and spread into the neighboring areas, which is the characteristic of a malignant tumor. Since CNNs are feature extractors, and deeper layers of the network operate in increasingly abstract spaces, the activation maps generated by the Grad-CAM method provide useful insights into which features had the most importance in determining the model’s final output class. These features are not just individual pixels, but a region of interest obtained by taking activation maps of deeper convolutional layers.

### 5.8. SHAP and SmoothGrad Results

**Figure 14:**
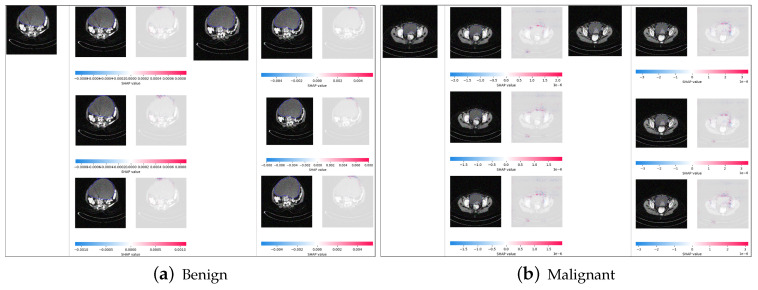
Results of SHAP and SmoothGrad on benign and malignant samples of cropped ovarian tumors data. Original images (**a**) and highlighted important pixels with the SHAP and SmoothGrad results (**b**).

The results for SHAP and SmoothGrad in Figure 14a,b show red–blue heatmaps on the benign and malignant images based on the SHAPley values. The red-colored pixels positively impact the model’s final prediction output, by increasing the model’s confidence for the final predicted class. The blue-colored pixels negatively impact the model’s final prediction output, by decreasing the model’s confidence for the final predicted class, benign or malignant, as shown in the figures. We see that the red pixels are mostly concentrated around the boundary and neighboring region of the tumor, indicating that the shape and localization of the tumor, as well as the ability to spread to the neighboring regions, is taken into consideration by the ResNet60 model for the classification.

### 5.9. Comparison of the Explainable AI Results

It is observed that LIME can identify the superpixels responsible for the final classification output, as well as the neighboring pixels positively and negatively impacting the classification, which proves that the shape and area of concentration of the tumor is a crucial factor for the classification. The Saliency Map highlights the region of interest of the image responsible for the classification output. We observe that this region of interest is also mostly concentrated around the edge and immediate neighboring region of the tumor, which proves that these play a significant role in the final classification output. The Grad-CAM results highlight the boundary of the tumor and its adjacent regions, indicating, once again, that these play a crucial role in the classification of the tumors as benign or malignant. The red–blue heatmap outputs for SHAP and SmoothGrad indicate that the red pixels increase the model’s confidence towards the final predicted output, and these pixels are mostly considered around the boundary and neighboring area of the tumor, thus re-affirming that the shape, localization, and ability of the tumor to spread to the neighboring areas have been given the most importance by the ResNet60 model for generating the final classification output. The heatmaps generated by the Occlusion Analysis attribution technique indicate that the shape and neighboring region of the tumor are more important towards the final output than the other input dimensions. Although Occlusion Analysis is very efficient at determining the marginal effect of each input dimension if the dimensions are independent, it has a high computational complexity, since we have to evaluate the model for each of the perturbed inputs, and if the input image has a higher size, the time required to generate the heatmaps increases manyfold.

The Explainable AI methods used in this paper for interpreting the classification results are qualitative in nature. They highlight the pixels or regions of interest in the image, which are considered as most significant by the ResNet60 classifier for classification of ovarian tumors as benign or malignant. One of the limitations of these methods is not being able to provide a quantitative explanation or metric for interpretation. There is a lot of research scope in designing metrics for higher-level feature importance measures, such as size, shape, boundary, localization, etc. The results of LIME showed that the model has given importance to the boundary and shape of the tumor, which is an important factor to consider for the classification of benign from malignant images. Results of the Saliency Map show the region of interest that the model has focused on the most for the generation of the output. The Grad-CAM results show that the model has given the most importance to the neighboring areas of the tumor as well as the shape and boundary of the tumor for distinguishing a benign tumor from a malignant one.

### 5.10. Scope of Future Work

There is a lot of scope for utilizing gradient-based approaches and feature-importance techniques for explaining deep neural networks better. As a next step, the deconvolution and guided backpropagation techniques can be implemented on the dataset of ovarian images for better interpretation. PatternNet, a newly emerging deep neural network architecture, is being used for identifying and visualizing patterns in the image data, which can explain the classification results of convolutional neural networks on these images. Mining visual patterns in images has the potential to reveal useful information about the intrinsic properties of objects and images, which influence neural networks to arrive at a certain classification decision.

## 6. Conclusions

In this paper, we have implemented several state-of-the-art techniques for the classification of tumors in the ovary as benign or malignant, and observed that a custom ResNet60 (or an enhanced ResNet50) architecture yields the best results on the test dataset. Thus, we have evaluated and interpreted the results of the modified ResNet50 classifier on a dataset of benign and malignant ovarian tumor images using different types of explainable AI techniques. Since the model has a 97.5% accuracy on the test dataset, the results of the explainable AI techniques show that the custom ResNet50 model has given importance to the correct features and regions of interest in determining whether an image of an ovarian tumor is a benign or malignant one. Thus, with the help of these Explainable AI methods, we interpreted the results of the proposed model, and understood which features or regions of interest it has given importance for generating the final classification output.

The source code for CNN architectures and Explainable AI methods in this paper are available at: https://github.com/srirupa-guha/Explainable_AI_Ovarian_Tumor_Classification.git (accessed on 30 April 2024).

## Figures and Tables

**Table 1:** Comparative analysis of existing work for tumor classification using CNNs.

References	Problem Statement	Dataset Used	Algorithms	Performance	Comparative Analysis with Proposed System
Jan, Y. T et al. [3]	Classification of ovarian tumors into benign and malignant using a proposed AI model	CT scanned images of 185 ovarian tumors from 149 patients	Ensemble AI model	Accuracy: 82%, Specificity: 89%, Sensitivity: 68%	The authors have achieved an accuracy of 82% for classification on this dataset, which is higher than the existing system used by junior radiologists. In our existing work, we used a dataset of 53 patients from a hospital in India. The results show a higher accuracy of 97.5% on the test dataset for classification using the proposed modified ResNet50 model.
Gupta, M et al. [6]	Brain tumor classification using CNN	MRI scanned images from UCI repository available publicly	Custom CNN and VGG16	Custom CNN achieved 100% accuracy, while VGG16 achieved 96% accuracy	The authors have trained a highly accurate custom CNN model for brain tumor classification, as well as tested a pre-trained VGG16 model. In our paper, as compared to VGG16 and other SOTA architectures, the proposed modified ResNet50 achieves the highest accuracy of 97.5% on the ovarian tumor test dataset.
Yadav, S et al. [7]	Medical image classification using deep CNNs	Chest X-ray images classified as Normal and Pneumonia	Custom CNN and VGG16	Custom CNN achieved 88.3% accuracy, while VGG16 achieved 92.4% accuracy	The authors have implemented custom CNN and VGG16 on a chest X-ray images dataset, and achieved an accuracy of greater than 90% for VGG16. In our paper, as compared to VGG16, the proposed modified ResNet50 achieves higher classification performance on the ovarian tumor dataset.

**Table 2:** Comparative analysis of existing work for explainability of CNNs.

References	Problem Statement	Dataset Used	Algorithms	Comparative Analysis with Proposed System
An, J. et al. [18], Soltani, S. et al. [35]	Explainability of CNN model output using LIME explanations	Wine Quality Dataset, Pima Indians Diabetes dataset, publicly available dataset comprising 14,380 images of 66 bird species from Flickr	LIME	An, J. et al. [18] used LIME for explaining feature importance in a binary classification problem using numerical and categorical attributes. Soltani, S. et al. [35] have evaluated the performance of LIME-generated explanations on bird species images, and evaluated the performance of the bird species classification explanation by conducting study trials that employed expert bird-watchers, who are able to distinguish bird species through specific characteristics and distinguishing features. In our research work, the concept of LIME or local explanations was extended to identify the characteristics of a tumors that cause it to be benign or malignant.
Alqaraawi, A. et al. [19]	Explainability of CNN models using Saliency Map and evaluation of performance	ILSVRC-2013 test dataset, PASCAL VOC dataset, COCO dataset	Saliency Map	The authors of these research papers presented class saliency as a method for the explainability of CNN output, as well as evaluation of the performance of these methods using scores for the saliency features, class sensitivity, and stability of the predicted confidence. In our research work, we have implemented the Saliency Map on the ovarian tumor dataset by constructing class Saliency Maps as described in the papers. However, evaluation of the explanation is beyond the scope of this paper, and is a prospective topic for continuation of this work.
Selvaraju, R. R. et al. [23], Fu, R. et al. [25], Cao, Q. H. et al. [24]	Explainability of CNN classification output using Class Activation Mapping (Grad-CAM, Segmentation CAM)	ImageNet Large Scale Visual Recognition Challenge (ILSVRC) data set, publicly available dog and cat binary classification dataset	Grad-CAM	Selvaraju, R. R. et al. [23] proposed the Gradient Class Activation Mapping (Grad-CAM), which highlights the regions of interest in an image for a target class based on the gradients. Fu, R. et al. [25] have provided an enhanced version of the Grad-CAM, called XGrad-CAM, that takes into account the two axioms’ sensitivity and conservation for evaluating the Grad-CAM explanations. Cao, Q. H. et al. [24] have proposed the Segmentation - Class Activation Mapping (SeCAM), which combines the capabilities of both LIME and Grad-CAM.
Resta, M. et al. [22]	Explainability using Occlusion Analysis for biomedical dataset	Cuff-Less Blood Pressure Estimation Data Set (CBPEDS) dataset pertaining to physiological signals, the Combined measurement of ECG, Breathing and Seismocardiograms Database (CEBSDB), and the PTB Diagnostic ECG Database (PTBDB)	Occlusion Analysis	The authors have proposed occlusion-based explanations that determine the effect of each input feature on the final classification output. A similar approach of occlusion-based experiments were conducted on the ovarian tumor dataset in our study.
Smilkov, D. et al. [34]	Explainability of CNN output using SmoothGrad	ILSVRC-2013 dataset	SmoothGrad	The authors have proposed the SmoothGrad method, an enhancement of the sensitivity maps based on gradients. In our paper, we have followed a similar approach to implement SmoothGrad for explanation of ResNet60 output on the ovarian tumor dataset.
Lundberg, S. M. et al. [27], Zacharias, J et al. [5]	Explainability and feature selection for classification using SHAP	Publicly available datasets: Taiwanese bankruptcy prediction, German credit, diabetes, credit card fraud detection, Spam base, breast cancer Wisconsin (diagnostic)	SHAP	The authors have demonstrated a method to utilize SHAP for eliminating less important features before training the classification model. The feature importance scores generated by the SHAP model were used to select the top features (selected by the user through elimination) important for classification. However, the features considered here are numerical or categorical features. In our paper, we have demonstrated the use of SHAP on the ovarian tumor dataset to highlight the important pixels. Since the input dataset comprises images, the highlighted pixels qualitatively present an idea of the important features considered by the model.
Singh, A et al. [14], Van der Velden, B. H et al. [15], Linardatos, P. et al. [16], Shivhare, I. et al. [31]	Comparative studies demonstrating the use of explainable AI methods in the medical imaging domain	Medical imaging datasets like X-ray, CT-scan, etc.	Backpropagation- based approaches like backpropagation, deconvolution, guided backpropagation, Class Activation Mapping (CAM), Grad-CAM, LRP, SHAP, Trainable attention, Occlusion sensitivity, LIME, Textual Explanation, Testing with Concept Activation Vectors (TCAV)	The authors discussed attribution-based, occlusion-based and backpropagation-based explainability methods that can be used for a variety of datasets in the medical imaging domain, including X-rays, CT-scans, breast imaging, and skin imaging. We have used backpropagation, activation mapping, SHAPley features importance values, and local explanations using LIME and occlusion analysis in our existing work for a qualitative analysis of ovarian tumor classification. Apart from the above methods, the authors also explored the possibility of attention-based methods and concept vectors, textual justification, intrinsic explainability, etc. as a future scope to this research domain.
Bach, S et al. [28], Ishikawa, S. N. et al. [30], Montavon, G. et al. [32], Vermeire, T. et al. [38], Shrikumar, A. et al. [33], Wu, B. et al. [41]	Other explainability methods, apart from the ones mentioned above	MNIST dataset, remote sensing image dataset from the Sentinel-2 satellite, ILSVRC dataset, DNA genome sequence dataset, ImageNet-1K, Cifar-100	Pixel-wise explanations using layer-wise propagation, example-based explainable AI, deep Taylor decomposition, evidence counterfactual, DeepLift, channel and spatial attention model	The authors in these research papers proposed further methods to explain CNN predictions using pixel contributions, example-based AI, decomposition of deep neural networks, counterfactual explanations, deep learning-based feature selection and attention models for the explanation of CNN classification models. These methods have not been implemented in our paper, and provide a prospect for expansion of the scope of our future research by implementing these on ovarian tumor dataset.

**Table 3:** Dataset distribution: number of images across train, validation and test datasets.

Train		Validation		Test	
Benign	Malignant	Benign	Malignant	Benign	Malignant
2633	1011	1128	433	376	144

**Table 4:** Proposed ResNet60 (enhanced ResNet50) architecture.

Stage	Layers	Conv1 Filters	Conv2 Filters	Conv3 Filters	TotalConv Filters	Stride	Padding and ReLU	Batch Norm
1	Conv	64	64	256	384	(2, 2)	Valid	Yes
1	Identity (x2)	64	64	256	384	(1, 1)	Valid	Yes
2	Conv	128	128	512	768	(2, 2)	Valid	Yes
2	Identity (x3)	128	128	512	768	(1, 1)	Valid	Yes
3	Conv	256	256	1024	1536	(2. 2)	Valid	Yes
3	Identity (x5)	256	256	1024	1536	(1, 1)	Valid	Yes
4	Conv	512	512	2048	3072	(2, 2)	Valid	Yes
4	Identity (x2)	512	512	2048	3072	(1, 1)	Valid	Yes
5	Conv	512	512	2048	3072	(1, 1)	Valid	Yes
5	Identity (x2)	512	512	2048	3072	(1, 1)	Valid	Yes
6	Conv	1024	1024	4096	6144	(2, 2)	Valid	Yes
6	Identity (x2)	1024	1024	4096	6144	(1, 1)	Valid	Yes

**Table 5:** Hyperparameters of architectures implemented for classification.

Architecture	Input Image Size	Optimizer	Learning Rate	Batch Size	Maximum Number of Filters	Filter Size	Activation Function	Pooling Layers	Flatten Layer	Padding	Strides	Epochs	Steps per Epoch	Validation Steps	Loss
GoogLeNet	224 × 224×3	Adam	0.001	64	192	3 × 3	ReLU (Softmax for final layer)	Max Pooling, Average Pooling	Flatten	Same	2	200	10	5	Sparse Categorical Cross Entropy
Inception v4	224 × 224×3	Adam	0.0007	64	192	3 × 3	ReLU (Softmax for final layer)	Max Pooling, Average Pooling	Flatten	Same	2	150	10	5	Sparse Categorical Cross Entropy
VGG16	224 × 224×3	Adam	0.0005	64	512	3 × 3	ReLU (Softmax for final layer)	Max Pooling, Average Pooling	Flatten	Same	2	100	10	5	Binary Cross Entropy
VGG19	224 × 224×3	Adam	0.001	64	256	3 × 3	ReLU (Softmax for final layer)	Max Pooling, Average Pooling	Flatten	Same	2	150	10	5	Binary Cross Entropy
ResNet16	224 × 224×3	Adam	0.001	64	256	3 × 3	ReLU (Softmax for final layer)	Max Pooling, Average Pooling	Flatten	Same	2	200	10	5	Binary Cross Entropy
ResNeXt	224 × 224×3	Adam	0.001	64	512	3 × 3	ReLU (Softmax for final layer)	Max Pooling, Average Pooling	Flatten	Same	2	200	10	5	Binary Cross Entropy
Inception-ResNet	224 × 224×3	Adam	0.001	64	256	3 × 3	ReLU (Softmax for final layer)	Max Pooling, Average Pooling	Flatten	Same	2	200	10	5	Sparse Categorical Cross Entropy
Xception	224 × 224×3	Adam	0.001	64	728	3 × 3	ReLU (Softmax for final layer)	Max Pooling, Average Pooling	Flatten	Same	2	200	10	5	Sparse Categorical Cross Entropy
ResNet50	224 × 224×3	Adam	0.001	64	128	3 × 3	ReLU (Softmax for final layer)	Max Pooling, Average Pooling	Flatten	Same	2	200	10	5	Binary Cross Entropy
ResNet60 (enhanced ResNet50)	224 × 224×3	Adam	0.001	64	1024	3 × 3	ReLU (Softmax for final layer)	Max Pooling, Average Pooling	Flatten	Same	2	200	10	5	Binary Cross Entropy
EfficientNetB0	224 × 224×3	Adam	0.001	64	512	3 × 3	ReLU (Softmax for final layer)	Max Pooling, Average Pooling	Flatten	Same	2	150	10	5	Binary Cross Entropy
DenseNet121	224 × 224×3	Adam	0.001	64	1024	3 × 3	ReLU (Softmax for final layer)	Max Pooling, Average Pooling	Flatten	Same	2	200	10	5	Binary Cross Entropy

**Table 6:** Comparison of model performances on the CT Scan dataset for train and test.

Model Name	Variant Name	Train Accuracy	Train Loss	Test Accuracy	Test Loss
Inception	GoogLeNet (Inception v1)	91.2%	0.24	92.5%	0.25
	Inception v4	93.8%	0.12	80%	42.60
	Xception	93.75%	0.17	95%	0.07
	Inception-ResNet	97.5%	0.09	95%	0.09
VGG	VGG 16	71.8%	2.46	72.5%	2.42
	VGG 19	74.4%	0.56	74.37%	0.57
ResNet	ResNet16	98.75%	0.03	92.5%	0.17
	ResNeXt	95%	0.02	90%	0.40
	ResNet 50	96.2%	0.16	90%	0.52
	ResNet60 (proposed)	95%	0.19	97.5%	0.14
EfficientNet	EfficientNet B0	71.8%	0.60	72.5%	0.58
DenseNet	Densenet 121	70%	2.25	70.63%	0.54

**Table 7:** Classification metrics for enhanced ResNet50 (ResNet60).

Accuracy	Precision	Recall (TPR)	F1-Score	Specificity (TNR)	FPR	FNR	Error
97.5%	96.66%	100%	98.30%	90.97%	9.03%	0%	2.5%

## Data Availability

The dataset is available in the same GitHub repository: https://github.com/srirupa-guha/Explainable_AI_Ovarian_Tumor_Classification/tree/main/Annotated_Cropped_Dataset.
